# Existing antiviral options against SARS-CoV-2 replication in COVID-19 patients

**DOI:** 10.2217/fmb-2020-0120

**Published:** 2021-01-06

**Authors:** Reza Ghanbari, Ali Teimoori, Anahita Sadeghi, Ashraf Mohamadkhani, Sama Rezasoltani, Ebrahim Asadi, Abolghasem Jouyban, Susan CJ Sumner

**Affiliations:** ^1^Digestive Oncology Research Center, Digestive Diseases Research Institute, Tehran University of Medical Science, Tehran 1411713135, Iran; ^2^Department of Virology, Faculty of Medicine, Hamadan University of Medical Sciences, Hamadan 65178-38678, Iran; ^3^Foodborne & Waterborne Diseases Research Center, Research Institute for Gastroenterology & Liver Diseases, Shahid Beheshti University of Medical Sciences, Tehran 1985717411, Iran; ^4^Department of Veterinary Biomedical Sciences, University of Saskatchewan, Saskatoon, SK, S7N 5B4, Canada; ^5^Pharmaceutical Analysis Research Center & Faculty of Pharmacy, Tabriz University of Medical Sciences, Tabriz 5166/1573, Iran; ^6^Department of Nutrition, Nutrition Research Institute, University of North Carolina at Chapel Hill, Chapel Hill, NC 28081, USA

**Keywords:** antiviral drugs, COVID-19, drug repurposing, SARS-CoV-2

## Abstract

COVID-19 caused by SARS-CoV-2, is an international concern. This infection requires urgent efforts to develop new antiviral compounds. To date, no specific drug in controlling this disease has been identified. Developing the new treatment is usually time consuming, therefore using the repurposing broad-spectrum antiviral drugs could be an effective strategy to respond immediately. In this review, a number of broad-spectrum antivirals with potential efficacy to inhibit the virus replication via targeting the virus spike protein (S protein), RNA-dependent RNA polymerase (RdRp), 3-chymotrypsin-like protease (3CLpro) and papain-like protease (PLpro) that are critical in the pathogenesis and life cycle of coronavirus, have been evaluated as possible treatment options against SARS-CoV-2 in COVID-19 patients.

COVID-19 is a public health emergency and international concern which is a communicable infectious disease caused by SARS-CoV-2 [1]. This contagious disease that has become to a global dilemma was first identified in Wuhan City, Hubei Province, China, on December 2019 [2], and recognized as a pandemic by the WHO in March 2020 [3]. According to the WHO data, as of 15 August 2020, over 21 million laboratory confirmed COVID-19 cases have been reported with associated more than 755,000 deaths worldwide, and the numbers of infection and death are still increasing [4].

SARS-CoV-2 is an enveloped, positive-sense ssRNA (+ssRNA) virus belonging to the family of Coronaviridae [5]. This novel virus is the seventh known coronavirus, after HCoV-229E, HCoV-NL63, HCoV-OC43, HCoV-HKU1, MERS-CoV and SARS-CoV, with the ability to infect humans [6].

To date, no specific drug or vaccine to inhibit and control of SARS-CoV-2 in COVID-19 patients has been identified [7]. Due to the rapid and global outbreak of the SARS-CoV-2, as well as its lethal pneumonia in some patients, immediate identification of effective treatments is vital. Efforts are underway to develop new precaution and treatment strategies; however, it may take months or even years to evaluate the safety and clinical efficacy against patients with COVID-19 [8].

Currently, due to urgent needs to control this pandemic infection, therapeutic options include use of existing antiviral drugs that have potential effects on replication of this novel emerging coronavirus [9]. Several broad-spectrum existing drugs that have been used in patients infected with other similar viruses, are being evaluated in numerous *in vitro* and *in vivo* studies as potential treatments against the SARS-CoV-2 [10]. This paper provides a review of broad-spectrum antivirals that have potential efficacy against SARS-CoV-2 through targeting the structural and nonstructural proteins of the virus, which are critical in the replication.

## Characterizations & replication cycle of SARS-CoV-2

**Figure 1.**
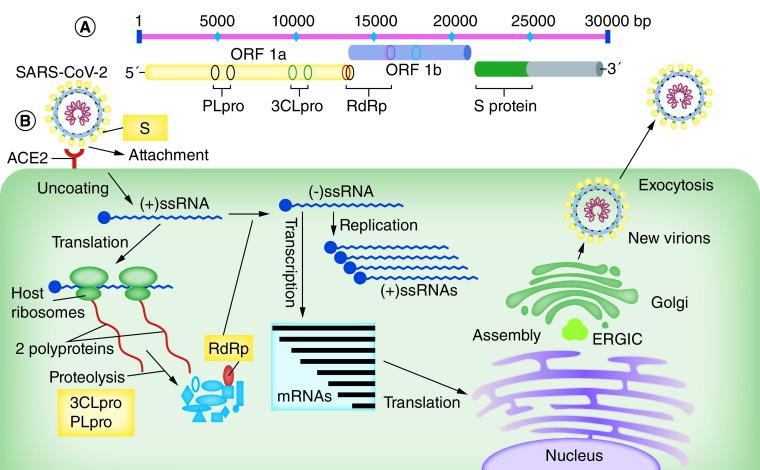
The genome organization and replication cycle of the SARS-CoV-2. **(A)** Genome analysis of SARS-CoV-2 reveals that the genome length is approximately 30 kb with several ORFs. Viral structural proteins such as 3CLpro, PLpro and RdRp are encoded from the two main ORFs. **(B)** Coronavirus infection begins with the attachment of the spike protein (S) with its receptor on the host cell’s surface (ACE2). Following adsorption and uncoating of the virus inside the host cells with an endosomal pathway, and releasing the NC to the cytoplasm, the viral ssRNA genome attaches to the host ribosomes to produce two polyproteins that are subsequently cleaved (in a proteolysis pathway) into smaller components, by host and viral proteases including 3CLpro and PLpro. The RdRp is involved in virus genome replication and also in viral mRNAs and their relevant viral proteins production. Then assembling of viral genome and viral proteins into new virions occurs in the host cell ERGIC. Eventually, new virions are transmitted outside the cells through exocytosis. 3CLpro: 3-Chymotrypsin-like protease; ACE2: Angiotensin-converting enzyme 2; ERGIC: Endoplasmic reticulum-Golgi intermediate compartment; NC: Nucleocapsid; ORF: Open-reading frame; PLpro: Papain-like protease; RdRp: RNA-dependent RNA polymerase; S: Spike protein; SARS-CoV-2: Severe acute respiratory syndrome coronavirus 2.

SARS-CoV-2 belongs to beta-coronavirus genus that also contains the SARS-CoV (caused the 2002 SARS outbreak in China) and MERS-CoV (caused the Middle East Respiratory Syndrome outbreak beginning in Jordan in 2012) [5]. The genome length of SARS-CoV-2 is approximately 30,000 bases with 10–14 open-reading frames (ORF) encoding 24–27 viral structural and nonstructural proteins (Figure 1A) [11,12].

The entry of the current coronavirus SARS-CoV-2 into the host cells is via interaction between the virus large surface glycoproteins (S protein) and the host cells surface receptors called angiotensin-converting enzyme 2 (ACE2) [13]. Current evidence revealed that SARS-CoV-2 virus S protein requires to be activated by cellular protease. Transmembrane protease serine 2 (TMPRSS2), a cellular surface serine protease, with effect on the S protein causes it to become fusion competent [14].

After virus adsorption and uncoating, two viral polyproteins are produced through host cell’s ribosomes. By using the host and virus proteases including the 3CLpro and PLpro, these polyproteins eventually are broken down into smaller viral nonstructural proteins such as RdRp. RdRp, a main enzyme encoded by the virus genome, is used for the replication of the virus RNA genome as well as producing the subgenomic mRNAs via discontinuous transcription [15]. These mRNAs subsequently produce viral structural proteins, during the translation process. Viral proteins and genome RNA would subsequently be assembled into new virions in the ERGIC, and then transported via smooth-wall vesicles to out of cells through exocytosis (Figure 1B).

The genome of SARS-CoV-2, similar to other species in this family, encodes structural proteins, such as spike (S), nucleocapsid (N), matrix (M) and envelope (E); and nonstructural proteins, such as RdRp, 3CLpro and PLpro. The virus surface glycoprotein (spike protein) is an essential protein for viral entry into the host cells and these nonstructural proteins are the key enzymes in the virus life cycle and their proliferation [15].

With the availability of the complete genomic sequence of the novel coronavirus, it is found that there is approximately 80% sequence identity between the SARS-CoV-2 and SARS-CoV [16]. Furthermore, it has been reported that the coding regions of S protein and also main nonstructural proteins including RdRp, 3CLpro and PLpro are much conserved with high similarity (>90% sequence identity) across the SARS-CoV-2 and SARS-CoV [7,16], especially in their functional areas and active sites [17,18]. Structural analysis revealed that there is more than 70% amino acids sequences identity in the active site of S protein, receptor-binding domain, which is essential for binding to ACE2, between SARS-CoV-2 and SARS-CoV [19,20].

## SARS-CoV-2 cell entry as promising target

Entry of the SARS-CoV-2 into target cells is facilitated by interaction between the viral spike glycoproteins as its major structural protein and host cell membrane receptor called ACE2 [13]. Spike protein (S) of SARS-CoV-2 and cell surface protease (TMPRSS2) mediate membrane virus fusion at the host cells. The S protein is cleaved into two functional fragments, S1 and S2, by a host cell-derived protease (furin-like protease). S1 subunit is responsible for binding to ACE2 and S2 subsequently will be cleaved by a cell surface protease called TMPRSS2. This process is necessary for virus–host cell membrane fusion [14]. Therefore, antiviral drugs with potential ability to inhibit the virus attachment and entry via targeting the S protein and TMPRSS2 could block the virus replication cycle and can be considered as a treatment strategies in COVID-19 patients.

Griffithsin is an alga-derived lectin with a broad-spectrum antiviral activity through viral entry inhibition. In both *in vitro* and *in vivo* studies, it has been demonstrated that griffithsin’s identical carbohydrate domains can bind to the specific oligosaccharides on the envelope of viral glycoproteins [21]. The efficacy of this compound is through attachment to the various viral surface glycoproteins, such as HIV glycoprotein 120 and SARS-CoV spike glycoprotein, has been shown in previous studies [22,23]. In several previous studies, griffithsin also demonstrated an *in vitro* activity against SARS-CoV and MERS-CoV [24–26]. Due to the high homology of SARS-CoV spike proteins with the novel identified coronavirus SARS-CoV-2 [27], the potency of griffithsin should be evaluated as a main candidate antiviral drug in treatment of COVID-19 patients.

Nafamostat, a drug used to treat acute pancreatitis, has been shown to control the activity of MERS-CoV by inhibiting the host protease TMPRSS2 that is required for S protein priming and virus membrane fusion entry to the host cells [28]. A recent report suggests that nafamostat has a potential efficacy to inhibit the membrane fusion of SARS-CoV-2 with host cells [29], and therefore might constitute a treatment option for patients with COVID-19.

## Antiviral options by targeting the SARS-CoV-2 protease

The critical step in the intracellular life cycle of SARS-CoV-2 and production of new virion particles is breakdown of viral polyproteins. This cleavage pathway depends upon viral protease including 3CLpro and PLpro [15]. Sequence alignment revealed a high sequence identity among the proteases of SARS-CoV-2 and SARS-CoV [16,30]. Accumulating evidence has demonstrated that SARS-CoV-2 proteases could be considered as a promising target for treatment of COVID-19 patients [31,32]. Disulfiram, lopinavir/ritonavir, danoprevir and nelfinavir as predeveloped antiviral drugs can inhibit the replication of wide range of viruses through a specific binding to viral proteases and blocking the proteolytic cleavage of viral protein precursors.

Disulfiram, a supportive drug in treatment of chronic alcoholism via acetaldehyde dehydrogenase inhibition [33], has been studied experimentally as a potential treatment for cancers [34] and latent HIV infection [35]. According to a study in 2018, disulfiram was found has a demonstrated ability to act as a competitive inhibitor of SARS-CoV as well as an allosteric inhibitor of MERS-CoV PLpro [36], however, no clinical data are available. Evidences suggest that this drug can inhibit the viral polyprotein cleavage through targeting the Zn-bound cysteines in PLpro, nsp10 and nsp13, and also catalytic cysteines in PLpro and 3CLpro [37,38]. Labile ZN sites have been identified to be present in PLpro, NSP10 and NSP13 of SARS and SARS-CoV-2 and due to their critical structural and functional role can be considered as targets for ejecting ZN^2+^ drugs such as disulfiram [39]. NSP10, by stimulating the methyltransferase activity and NSP13 by its helicase and 5′-triphosphatase activity, are important nonstructural proteins in the viral replication cycle [40]. Amino acid sequence alignment between SARS-CoV and SARS-CoV-2 have shown that there are 99.3 and 100% sequence similarity in NSP10 and NSP13 of these two viruses [40].

Lopinavir is an approved antiviral drug that is used to treat HIV infection by inhibiting the viral protease [41] and ritonavir, another approved antiretroviral drug from the protease inhibitor class, is widely used as a booster for other protease inhibitor drugs [42]. The combination of lopinavir and ritonavir (Kaletra) is commonly used for antiviral experiments as an approved single drug, to increase the half-life of lopinavir for longer effects [43]. In a recent computational approach to evaluate existing drug’s efficacy against SARS-CoV-2, Kaletra has been identified as having a high binding affinity for the pocket site of the 3CLpro enzyme in SARS-CoV and SARS-CoV-2, and may act as an inhibitor for viral replication [44]. In addition, the beneficial effects of kaletra on SARS-CoV and MERS-CoV infection have previously been observed in several *in vitro* and animal studies [45–47]; however, its efficacy on these viruses in human studies has not yet been well characterized.

Although evidence suggests that this antiviral drug potentially could control the SARS-CoV-2 infection via inhibition of viral proteases, a resent randomized, controlled, open-label trial study on 199 hospitalized patients with severe COVID-19, did not demonstrate benefit with kaletra treatment [48]. Therefore, according to the available data, it is complicated to assess whether this drug is efficient in treatment of COVID-19 patients, either as monotherapy or in combination with other drugs and whether or not its efficacy against SARS-CoV-2 replication should be confirm in future trials.

Furthermore, in a recent molecular-docking investigation performed by Xu *et al.*, Nelfinavir, an approved antiretroviral drug, was identified as a potential inhibitor against the 3CLpro of SARS-CoV-2 [49]. This HIV-1 protease inhibitor has been previously reported in an *in vitro* study to strongly inhibit the replication of the SARS-CoV, and reduce the induced cytopathic effect of the virus infection [50]. Nelfinavir and lopinavir were concluded to represent potential treatment options for COVID19 by targeting the 3CLpro of SARS-CoV-2, in another recent molecular docking study [51]. Additionally, in several cell culture models of SARS-CoV-2, nelfinavir reveals the potent to inhibit replication of SARS-CoV-2 and might be a potential candidate drug for the treatment of COVID-19 patients [52–54]. Therefore, based on its high potency against SARS-CoV-2, nelfinavir deserves further exploration as potential inhibitor of SARS-CoV-2 infection in patients with COVID-19.

Danoprevir (Ganovo), an oral Hepatitis C virus NS3 serine protease inhibitor, has been already approved in China to treat the noncirrhotic genotype 1b chronic hepatitis C [55,56]. Due to structural and functional similarity between HCV NS3 protease and SARS-CoV-2 3CLpro [57] the HCV protease inhibitors such as danoprevir are hypothesized to have therapeutic potential against SARS-CoV-2 infection. Recently, in a small clinical study performed by Chen *et al.* [58], the therapeutic efficacy of danoprevir in combination with ritonavir as booster has been evaluated on 11 patients with COVID-19. All patients in this study recovered after a 4 to 12-days treatment with danoprevir boosted by ritonavir, and it was also reported that this treatment was safe and well tolerable in all participants [58].

## RNA-dependent RNA polymerase as primary target to inhibit SARS-CoV-2

One of the most important therapeutic targets for coronaviruses is the RdRp. This vital enzyme is highly conserved and shares common structure with positive-sense RNA viruses such as coronaviruses and HCV [37]. In addition, its active site is highly conserved with two consecutive aspartate residues that protrude from a beta-turn structure [59].

This class of existing drugs is actually nucleotide and nucleoside analogs in the form of adenine and guanine derivatives. These nucleotide analog drugs can inhibit the viral genome replication and transcription via targeting the viral RdRp, in wide range of RNA viruses [60]. RdRp plays a central role in the transcription and replication of SARS-CoV-2 RNA genome, and appears to be a primary target for the nucleotide analog antiviral inhibitors [61].

Favipiravir (Avigan, T-705) is a pyrazinecarboxamide derivative and a guanine analog with activity against many RNA viruses such as influenza viruses, yellow fever virus, arenaviruses and enteroviruses [62,63]. Favipiravir can selectively inhibit the viral RdRp without inhibition of RNA or DNA synthesis in mammalian cells [62,63]. This flu drug has produced encouraging outcomes in treating the pneumonia caused by SARS-CoV-2 in two clinical trials in China involving 340 patients [10,64]. Furthermore, high concentrations of three nucleoside analogs (including ribavirin, penciclovir and favipiravir) were recently shown to reduce the SARS-CoV-2 infection [65]. In addition, in a recently open-label nonrandomized control study, the effects of favipiravir were evaluated in treatment of COVID-19 patients compared with the lopinavir/ritonavir and favipiravir has shown a significantly higher improvement rate with faster viral clearance and fewer side effects in patients with laboratory-confirmed COVID-19 [66].

Ribavirin, an approved drug to treat HCV and respiratory syncytial virus (RSV), is a guanosine analog with the ability to block viral RNA synthesis through viral mRNA-capping termination by inducing several mutations in the genome of the RNA viruses targeting the viral RdRp [67]. The efficacy of this drug has been evaluated for patients infected with SARS-CoV and MERS-CoV, although the severe side effects of ribavirin caused the limitation of use this kind of treatment in patients [67,68].

In an *in vitro* study regarding the evaluations of potential antiviral drugs against the SARS-CoV-2, antiviral effects of ribavirin against a clinical isolate of SARS-CoV-2 were 100-times less effective than remdesivir [65]. In a molecular docking experimental model, ribavirin and sofosbuvir were able to bind tightly to the RdRp of the SARS-CoV-2 virus, therefore suggesting possible efficacy of these two drugs in treatment of this novel coronavirus. However, the efficacy and safety of ribavirin and also its substantial toxicity in COVID-19 patients is not yet well understood, and requires further clinical experiments based on the poor *in vitro* and no animal or human data.

Remdesivir is another investigational intravenous broad-spectrum antiviral drug. This drug is actually an adenosine analog that can binds to the viral RdRp and acts as an RNA-chain terminator [69]. Remdesivir has been developed in response to the Ebola outbreak in West Africa from 2014 to 2016 [70]. Apart from the Ebola virus infection, remdesivir has also activity against a wide range of ssRNA viruses, such as SARS-CoV, MERS-CoV, Marburg and RSV [69].

Very recently, an *in vitro* study in vero E6 cells showed the efficacy for five US FDA-approved drugs including ribavirin, penciclovir, nitazoxanide, nafamostat and chloroquine as well as two well-known broad-spectrum antiviral drugs, remdesivir and favipiravir. These have been clinically evaluated against SARS-CoV-2 virus, and notably, remdesivir and chloroquine were able to potentially block this novel coronavirus infection [65].

Recently, after the worldwide outbreak of SARS-CoV-2, remdesivir was injected intravenously into a patient in the United States who was infected with SARS-CoV-2 and exhibited severe symptoms. The patient responded quickly to remdesivir [71]; however, such fast and effective improvement in this patients’ condition may be attributed to a strong immune system or other supportive treatments, and further studies are needed to understand the efficacy and potential side effects. Furthermore, the first Italian COVID-19 patient was successfully treated by using this drug on March 2020 [72].

According to a review on potential drugs against SARS-CoV-2 infection in COVID-19 patients, two Phase III clinical trials have been initiated to evaluate the efficacy and safety of remdesivir as a therapeutic option against COVID-19 infection [7]. Emerging clinical evidence obtained from *in vitro* studies against SARS-CoV-2 and *in vitro* and *in vivo* studies against related beta coronaviruses suggests that remdesivir could be a promising compound to treat severe COVID-19 patients, at least until the development of new specific compounds against the SARS-CoV-2 virus, and more in-depth studies are needed.

Galidesivir, an adenosine analog, also can act as a nucleoside RNA polymerase inhibitor that is able to restrict the viral replication process via premature termination of RNA transcription [73]. Galidesivir is used as an antiviral drug against the HCV infection and has been shown to antiviral activity against a wide range of RNA viruses such as SARS-CoV, MER-CoV, Ebola and Marburg [74]. Therefore, it may be considered as an interesting drug candidate for further development in treatment of the SARS-CoV-2 virus in COVID-19 patients with severe symptoms.

All existing antivirals with potential ability against SARS-CoV-2 replication that discussed in the present review are summarized in Table 1.

**Table 1.** Repurposing antivirals with potential ability to inhibit the SARS-CoV-2 replication via targeting the viral structural and nonstructural proteins.

Existing antivirals	Chemical structure	Primary use	Potential efficacy on other viruses	Mechanism of action	Study (year)	Ref.
Griffithsin	PubChem SID: 163672929	An alga-derived lectin against HIV	Broad-spectrum antiviral activity against HIV, SARS and MERS	Viral entry inhibition by attachment to the virus surface glycoproteins such as HIV gp120 and SARS-CoV-2 S protein	Lee *et al.* (2019) Fischer *et al.* (2019) Ziółkowska *et al.* (2006) Millet *et al.* (2016)	[22–25]
Nafamostat	PubChem CID: 441**3**	A drug for acute pancreatitis	Inhibition the MERS and SARS-CoV-2 life cycle	Inhibiting the host cells protease (TMPRSS2) and virus membrane fusion	Yamamoto *et al.* (2016)	[28]
Disulfiram	PubChem CID: 3117	A drug for chronic alcoholism	Potential treatment for HIV and inhibition ability for MERS and SARS-CoV-2	Inhibit the viral polyprotein cleavage	Lin *et al.* (2018) Anand *et al.* (2003) Lee *et al.* (2016)	[36–38]
Lopinavir/ritonavir (Kaletra)	PubChem CID: 92727 PubChem CID: 392622	A drug to treat HIV	Inhibit the replication of the SARS, MERS and SARS-CoV-2	Inhibiting the viral replication by high binding affinity to the viral protease	Chu *et al.* (2004) Yao *et al.* (2020) Cao *et al.* (2020)	[46–48]
Nelfinavir	PubChem CID: 64143	Approved antiretroviral drug against HIV-1	Inhibit the replication of the SARS and SARS-CoV-2	Potential inhibitor against the viral protease such as 3CLpro	Xu *et al.* (2020) Yamamoto *et al.* (2004) Ohashi *et al.* (2020)	[49,50,54]
Danoprevir	PubChem CID: 11285588	A drug for noncirrhotic genotype 1b HCV	Therapeutic potential against SARS-CoV-2	Potential inhibitor against the viral protease such as 3CLpro	Chen *et al.* (2020)	[58]
Favipiravir (Avigan)	**** PubChem CID: 492405	A guanine analog flu drug	Activity against many RNA viruses such as influenza virus, yellow fever virus, enterovirus, SARS-CoV-2, …	Selectively inhibit the viral RdRp via tightly binding to the RdRp	Furuta *et al.* (2009) Furuta *et al.* (2013) Cai *et al.* (2020)	[62,63,66]
Ribavirin	PubChem CID: 37542	An approved drug against HCV and RSV	Activity against of MERS and SARS-CoV-2	Inhibition the viral RNA synthesis by viral mRNA capping termination via targeting the viral RdRp	Stockman *et al.* (2006) Arabi *et al.* (2020)	[67,68]
Remdesivir	PubChem CID: 121304016	An adenosine analog developed in response to the Ebola virus	Broad-spectrum antiviral activity against a wide range of ssRNA viruses such as SARS, MERS, Marburg, RSV, SARS-CoV-2, etc.	Binds to the viral RdRp and acts as an RNA-chain terminator	Wang *et al.* (2020) Sheahan *et al.* (2017) Holshue *et al.* (2020)	[65,69,71]
Galidesivir	PubChem CID: 10445549	An adenosine analog developed in response to the HCV	Antiviral activity against a wide range of RNA viruses such as SARS-CoV, MER-CoV, Ebola and Marburg	Inhibition the viral RNA polymerase and premature termination of RNA transcription	Westover *et al.* (2018)	[74]

The chemical structures of existing antivirals provided in Table 1 are derived from ‘PubChem’ database [75]. PubChem CIDs and SID are available in Table 1.

CID: PubChem’s compound identifier; Flu: Influenza; HCV: Hepatitis C virus; RdRp: RNA-dependent RNA polymerase; RSV: Respiratory syncytial virus; SID: PubChem’s substance identifier; TMPRSS2: Transmembrane protease serine 2.

## Discussion

The global outbreak of novel highly contagious COVID-19 caused by SARS-CoV-2 has become a major public health issue and clinical threat to the general population worldwide. However, knowledge about the SARS-CoV-2 and its unknown disease remains limited. Due to urgent needs to prompt responses against this pandemic viral infection, developing effective therapeutic compounds is vital.

Therapeutic strategies for patients with COVID-19 infection are generally based on two categories considering their targets, including drugs that target the SARS-CoV-2 life cycle and drugs with efficacy on human immune system or host cells.

In general, three strategies can be used to identify efficient drugs against emerging viral infections such as SARS-CoV-2. These include: Developing novel exactly specific compounds using the genomic information and pathological characteristic of the virus. Many research groups around the world are currently working on developing novel antivirals against SARS-CoV-2, such as designing SARS-CoV-2 S protein-neuralizing antibodies [76] or specific synthetic peptides [77] that can prevent the virus attachment by targeting the S protein receptor-binding domain, or drawing drugs that disrupt the virus fusion via targeting the virus S2 subunit [78], or also survey the potential effects of siRNAs, antisense oligonucleotides or RNA aptamers with ability to bind to the SARS-CoV-2 RNA genome [79,80]. Although this approach can be clinically useful, considerable time will be needed evaluate its pharmacokinetic and pharmacodynamic properties as well as its side effects in animal and human trials. Molecular docking studies that rely on available molecular databases for the identification of drugs that can inhibit the virus replication cycle provide a means for rapid identification of potential drug candidates. The studies that have been performed by Peele *et al.* [81], Calligari *et al.* [82] and Yu *et al.* [17] on SARS-CoV-2 antiviral drugs discovery by using the *in vitro* molecular docking technologies are examples of this strategy. Testing existing broad-spectrum antiviral drugs that have previously been shown to have efficacy toward similar viruses. This worthwhile approach, due to its established safety records, understanding of metabolic characteristics, dosage used, potential efficacy and side effects, seems to be extremely worthwhile, and our main focus in this review has been on this strategy to evaluate the existing antivirals with potential efficacy against SARS-CoV-2 infection. For instance, Chen *et al.* conducted a clinical study by using a HCV protease inhibitor, danoprevir, to evaluate the effects of this existing antiviral in treatment of patients with COVID-19 [58]. In addition, two recently separate studies on remdesivir (an existing developed antiviral against Ebola virus) efficacy to inhibit the SARS-CoV-2 infection by Wang *et al.* [65] and Holshue *et al.* [71], are other examples of the third strategy. To control this novel pandemic severe respiratory illness, the experiences used to control similar epidemics infections such as SARS-CoV and MERS-CoV should be considered.

Numerous clinical trials are currently ongoing across the world in which large number of antivirals and immunomodulators are being investigated against COVID-19 patients. All of these attempts are aimed to decrease mortality and morbidity rate of this novel contagious disease until a specific drug or an effective vaccine is developed.

## Future perspective

Unfortunately, to date, there is no specific efficient treatment to inhibit the SARS-CoV-2 in patients with COVID-19 infection. Due to the rapid outbreak of the SARS-CoV-2 and mortality rate of this novel pandemic infection, urgent needs to control this highly contagious disease by developing new treatments or using the existing antivirals with potential ability to inhibit this virus replication are vital. Several clinical trials are possessing worldwide to find efficient therapeutic compounds against this novel virus through inhibition the virus replication cycle by targeting the structural and/or nonstructural proteins. In a review by Kumar *et al.* [32] on repurposing antivirals against SARS-CoV-2 in COVID-19 patients, extracellular vesicles (EVs) as a novel drug-delivery system have been evaluated. EVs are natural carriers for biological molecules such as DNA, RNA, miRNA or other small molecules. In this review, the authors have shown that encapsulated antiviral drugs by EVs as natural nanocarriers, have the ability to target specific tissues, which increase their efficacy and safety in COVID-19 patients [32]. The future goal is to complete clinical studies and find the most effective, safe, cost-saving and tolerable treatments, antiviral drugs and vaccines for COVID-19 infection.

**Executive summary**

**Introduction**

- SARS-CoV-2 is a member of Coronavirdae family that contain human and animal viruses that cause serious disease.
- SARS-CoV-2 has a large RNA genome approximate 30 kb encoding several structural and nonstructural proteins.
- The disease caused by SARS-CoV-2 called COVID-19 is the largest worldwide concern and the main health emergency in 2020.
- Urgent efforts to develop novel effective treatment, antivirals or efficient vaccines are vital to control this contagious disease.
- Comprehensive efforts are currently ongoing to identify specific drugs against SARS-CoV-2 may take months to evaluate their safety and clinical efficacy.
- Use of repurposing antivirals with potential ability to inhibit SARS-CoV-2 might be reasonable strategy against this novel coronavirus.

**Virus entry as promising target**

- Entry of the SARS-CoV-2 into target cells is facilitated by interaction between the virus spike protein (S protein) and host cell receptor called ACE2, as well as cell surface protease (TMPRSS2) mediates virus fusion to the host cells.
- Based on previous experience in virology, viral attachment and entry inhibitors can be considered as a treatment strategies in COVID-19 patients.
- Griffithsin, a lectin isolated from the red algae, can bind to the virus surface glycoproteins such as SARS-CoV S protein.
- Nafamostat can inhibit the membrane fusion of SARS-CoV-2 through inhibiting the host cells protease (TMPRSS2).

**Targeting the SARS-CoV-2 protease**

- One of the critical step in SARS-CoV-2 life cycle is cleavage of viral polyproteins by using the viral protease including 3-chymotrypsin-like protease (3CLpro) and papain-like protease (PLpro).
- Viral protease inhibitors can inhibit the virus replication via binding to viral proteases.
- Disulfiram, an antiviral for HIV, has inhibition ability for MERS and SARS-CoV-2 by inhibit the viral polyprotein cleavage.
- Lopinavir/ritonavir and nelfinavir that are originally antiretroviral drugs for HIV have a high binding affinity for the 3CLpro in SARS-CoV-2 and may act as an inhibitor for viral replication.
- An HCV protease inhibitor, danoprevir, may consider as a therapeutic antiviral against SARS-CoV-2 via binding to the viral 3CLpro.

**Targeting the SARS-CoV-2 RNA-dependent RNA polymerase**

- Nucleotide analog drugs can inhibit the viral replication cycle through targeting the viral RNA-dependent RNA polymerase (RdRp), which plays a central role in the transcription and replication of RNA genome.
- Favipiravir is a guanine analog with activity against many RNA viruses such as SARS-CoV-2 via inhibition the viral RdRp.
- Ribavirin and galidesivir, the originally antiviral drugs against the HCV, are able to bind to the RdRp of SARS-CoV-2 and inhibit the viral RNA synthesis.
- Remdesivir, an adenosine analog, a primary developed drug to treat the Ebola has a potential activity against a wide range of RNA viruses such as SARS-CoV-2 by binding to the viral RdRp and acts as an RNA-chain terminator. Remdesivir may be considered as an interesting antiviral candidate to treat the COVID-19 patients.
